# Class Address

**Published:** 1874-04

**Authors:** John. W Farmer

**Affiliations:** Virginia.


### ARTICLE III.

### *Class Address.*

Delivered at the *Thirty-fourth* Annual Commencement of the Baltimore College of Dental Surgery.

BY JOHN. W FARMER, M. D., OF VIRGINIA.

*Ladies and Gentlemen*:—We are here to celebrate the *thirty-fourth* annual commencement of the Baltimore College of Dental Surgery; and on the part of the graduating class, I am before you not only to extend to you a cordial welcome to this hall, but also the most heart-felt thanks of every member of the school for the kindly interest you have manifested in us by your presence here to night. It is indeed gratifying to us, that at the closing hour of our collegiate course we should be greeted with the smiles and floral demonstrations of so many kind friends and fair ladies of this most renowned city.

Our stay of a few short months among you has been sufficient to bind you to us with cords of friendship that can never be broken. We can take leave of you to-night with sincere reluctance, and shall carry to our homes the most grateful remembrance of your cordial hospitality and genuine friendship. Permit us in parting to assure you that we appreciate the high social advantages you have so cheerfully accorded us, and shall ever cherish for you the fondest recollections, and the most pleasant memories.

*Gentlemen of the Faculty:*—It is painful to realize that this is the last time we shall meet you as a class. The earnestness and zeal with which you have taught us, the patient self-denying sacrifices which you have ever been ready to make for us, and the anxious care and counsel you have bestowed, deeply impress us with your skill and ability as instructors, and induce us to believe that your kindly interest and solicitude will follow us beyond the precincts of our college life. We need not assure you that in all coming time we shall cherish for each and every one of you the most abiding friendship and affectionate regard.

Willingly would we linger to listen to your words of wisdom, and draw lessons of instruction from your patient and careful teaching. In this moment of painful parting, accept if you please, our most sincere and heartfelt thanks, both for the very many acts of kindness and courtesy which we have ever met at your hands, and for the able and efficient manner in which you have guided us in the arduous tasks we have had to perform.

*Gentlemen of the Graduating Class.*—It becomes my pleasant duty before we part to say a few words about the duties and responsibilities which await us, as members of the profession into whose ranks we are this night admitted.

Recognizing the imperative necessity of a thorough course of mental discipline to entitle its graduates to the just claim of members of a liberal profession, to the dignity of which our specialty has now risen, this College has embraced in its course besides the purely dental branches, as full instruction in several collateral sciences as is taught in the universities and best medical colleges in this country.

But neither professional nor literary institutions claim to send out their graduates as men of great learning and ripe scholarship at the time they confer their degrees. The great purpose of the long course of training to which they are subjected, is not so much mere acquisition of actual knowledge, as to teach them how to think for themselves; to unfold their capacities and powers of mind; to give them broad expanded views; to build up within them a *force* of thought which may be turned at will on any subject upon which they may be called to pass their judgment; to impart to them a concentration of attention, an accuracy of observation which will enable them to reduce complex subjects to their elements, and dive beneath effects to causes, and rise from particular facts to general truths.

The young graduate of medicine, after he has enjoyed all the best advantages of the lecture and the clinic, upon assuming the practice of his profession, finds that he is in possession of not much more than the theoretical part of it. No amount whatever of mere theory can put him at once at his ease at the sick bedside, and give him confidence, efficiency and success there.

The teachings of our specialty, I am aware has not been wholly theoretical; but we have been instructed as far as possible in the practical manipulating details of it, and still we must all feel that we are standing simply upon the threshold of a great work before us, with only the foundation laid, while the superstructure is yet to be erected. Let us strive to build substantially upon this foundation, and see to it that the fabric we rear shall in quality of material, in artistic execution, in symmetry of shape, and in style of architecture, correspond strictly with the plans and specifica tions that have been given to us.

Dentistry has but too recently been assigned to the elevated position which it now occupies, as a truly scientific pursuit to have engaged the laıge number of learned men, which has accumulated in the older professions. But from the first there have stood out among those who have been engaged in it, men who would have been ornaments in any position of life in which they might have been placed; men who labored under the greatest difficulties to develope it into a profession. The idea for a long time prevailed that it was a purely mechanical pursuit, and for the most part men without a great amount of culture, simply mechanics, were attracted to it. And not until more recently, has the fact been fully recognized that there is the same necessity for a thorough medical course to gratify the dental practitioner for the successful prosecution of his work, as should be possessed by the general surgeon ; and, furthermore, that it calls for the cultivation of æsthetics to a large extent ; that to be a good dentist, one should not only be a mechanic, but an artist in many respects of the highest type.

What we want in the profession now, and what we are happy to believe are being added yearly to it, are men of a high order of education and qualification ; men who are prepared to illustrate more fully the utility of our calling, by bringing to their aid the most liberal studies and largest improvements attainable by cultivating scientific pursuits, enlarging their views and reaching out in every direction for knowledge, which they can lay under contribution to it. Such a course of cultivation must necessarily contribute largely in many ways to the advancement of our science and art, by affording the needed aids and appliances in carrying forward the many improvements which are yet to be made in our specialty. The last few years have witnessed a progress in both the scientific and artistic departments wholly unprecedented, and I hazard nothing in predicting that during the next few years the developments will keep pace with the recent advancements. Why may we not aspire to assist in carrying them forward ?

*Fellow Classmates* :—It now only remains for me to add a single word of parting, and the duty which you have so kindly assigned me is performed.

We met here at the commencement of our college course as strangers ; together we have toiled through the college curriculum, and are now about to pronounce to each other the saddest of all our words, farewell. Memory loves to dwell on college days, and for each other I am sure we will cherish through all our coming lives the fondest recollections of our associations here. Together we have toiled long and patiently for the reward which we have this evening gained. We have received our diplomas, and enter now upon those higher walks of professional life, for whose steep ascents our careful disciplining, I trust, has somewhat prepared us. What incentives there are to stimulate us to noble exertions in the prosecution of the profession of our choice!

We carry with us the credit of our science and art, the reputation of our teachers and the name of our *alma mater*. As the custodians of these sacred truths, and with a firm resolve to do our whole duty at all times, in all places, and to all men, let us go forward with an abiding faith, that our success will be commensurate with the high obligations that are upon us.

And now ladies and gentlemen, professors and classmates, farewell.

---

